# Phenotype Prediction and Genome-Wide Association Study Using Deep Convolutional Neural Network of Soybean

**DOI:** 10.3389/fgene.2019.01091

**Published:** 2019-11-22

**Authors:** Yang Liu, Duolin Wang, Fei He, Juexin Wang, Trupti Joshi, Dong Xu

**Affiliations:** ^1^Institute of Data Science and Informatics, University of Missouri, Columbia, MO, United States; ^2^Department of Electrical Engineer and Computer Science, University of Missouri, Columbia, MO, United States; ^3^Christopher S. Bond Life Science Center, University of Missouri, Columbia, MO, United States; ^4^Department of Computer Science and Information Technology, Northeast Normal University, Changchun, China; ^5^Department of Health Management and Informatics, School of Medicine, University of Missouri, Columbia, MO, United States

**Keywords:** genomic selection, deep learning, genome-wide association study, soybean, genotype contribution

## Abstract

Genomic selection uses single-nucleotide polymorphisms (SNPs) to predict quantitative phenotypes for enhancing traits in breeding populations and has been widely used to increase breeding efficiency for plants and animals. Existing statistical methods rely on a prior distribution assumption of imputed genotype effects, which may not fit experimental datasets. Emerging deep learning technology could serve as a powerful machine learning tool to predict quantitative phenotypes without imputation and also to discover potential associated genotype markers efficiently. We propose a deep-learning framework using convolutional neural networks (CNNs) to predict the quantitative traits from SNPs and also to investigate genotype contributions to the trait using saliency maps. The missing values of SNPs are treated as a new genotype for the input of the deep learning model. We tested our framework on both simulation data and experimental datasets of soybean. The results show that the deep learning model can bypass the imputation of missing values and achieve more accurate results for predicting quantitative phenotypes than currently available other well-known statistical methods. It can also effectively and efficiently identify significant markers of SNPs and SNP combinations associated in genome-wide association study.

## Introduction

The marker-assisted selection (MAS) strategy has made significant improvements in phenotype prediction for quantitative traits in breeding, assuming that genotype markers have significant associations with their phenotypes. The genome-wide association study (GWAS) has also been applied to select those phenotype-associated genetic variants. Genomic selection (GS) is one type of MAS strategy, using single-nucleotide polymorphisms (SNPs) to predict breeding values (BVs) or quantitative phenotypes. The strategy has been widely applied in i) major crops (Jannink et al., 2010), such as soybeans [*Glycine max*], rice [*Oryza sativa*], and maize [*Zea mays*] (Zhao et al., 2012; Spindel et al., 2015; Xavier et al., 2018); ii) crops with long life cycles, such as oil palm [*Elaeis guineensis* Jacq.] (Cros et al., 2015) and domesticated animals like Holstein dairy cattle (Schaeffer 2006; Verbyla et al., 2009). Traditional statistical methods, such as the best linear unbiased prediction (BLUP), Bayesian A, B, Cπ, and Bayesian LASSO (BL) (Hayes and Goddard, 2001; Pérez et al., 2010; Endelman, 2011) have been widely utilized for modeling genotype effects and predicting phenotypes. These statistical methods usually assume that genotype random effects follow a prior distribution such as Gaussian, and the contribution of each genotype to the associated phenotype is considered as an independent feature. This prior assumption requires sufficiently large training samples to cover the overall population structure and to make it true. However, in practice, the individual genotype effect is unknown and may not strictly follow a certain distribution. In addition, SNPs may also have interactions with other SNPs that contribute to complex diseases or traits (Wang et al., 2015) as seen due to the epistasis effects.

Missing values in a genotype matrix represent another challenge for statistical methods, wherein these missing values are usually screened out during preprocessing or filled with values through imputation (Howie et al., 2009; Marchini and Howie 2010; Rutkoski et al., 2013). Imputation is a computational process for estimating missing values in genotypes from a template population. Several methods have been developed for genomic imputation with or without the reference genome information. The calculated mean, expectation–maximization (EM) algorithm is provided in the R package rrBLUP (Endelman 2011); random forest (RF) is provided in missForest (Stekhoven and Bühlmann, 2011), and a hidden Markov model (HMM)-based method is applied in BEAGLE (Browning et al., 2018) and MaCH (Li et al., 2010) with the reference genome. The imputation accuracy is highly dependent on observed non-missing genotypes and the missing rate of the whole population, which directly affects the performance of the phenotype prediction model (Rutkoski et al., 2013; Xavier et al., 2016). To develop a phenotype prediction model through statistical approaches, the genotype matrix is required to be imputed together and then divided into training and testing datasets for model training and testing. To some extent, the testing set is not totally independent from the training set, since the training set may contain genotypes estimated from the testing set under this circumstance. Inaccurate imputation methods may also introduce errors and uncertainty and further affect biomarker selection. Therefore, these imputation approaches may not be effective in inferring informative genetic markers hidden in the entire genome.

Recently, deep learning has been applied in computational biology (Angermueller et al., 2016), with the introduction of noncoding variant function prediction (Zhou and Troyanskaya, 2015), protein localization prediction (Alipanahi et al., 2015; Zhang N et al., 2018), protein secondary structure prediction (Spencer et al., 2015), and protein post-translational modification site prediction (Wang D et al., 2017; Wang et al., 2018). In genotype association studies, deep learning has also been used to identify SNP interactions (Uppu et al., 2016), classify genomic variants (Liang et al., 2016). DeepGS, an ensemble of convolutional neural network (CNN) (Krizhevsky et al., 2012) and rrBLUP have been used to predict phenotypes using imputed SNPs (Ma et al., 2018), and a simple dense neural network (DNN) is used on genotype-by-sequencing (GBS) data (Montesinos-López et al., 2018). For these phenotype prediction problems, CNN can capture spatial information from raw sequencing reads or genomic variants without feature engineering. To some extent, the CNN also resolves the local epistasis effect as the convolving process is considering interactions among neighboring SNPs within different ranges of the kernel window. However, the above deep learning methods have not effectively addressed the problem of missing values, and they all treat the deep learning models as black boxes without discussing the effective SNP markers. In particular, none of them have explored the internal features associated with the traits through attention mechanisms, which is an approach developed for visualization of the black box of deep learning architecture. The saliency map (Simonyan et al., 2013) of deep learning was first introduced for visualizing image features in classification and now plays a major role in image segmentation and image style transfer (Gatys et al., 2016). This strategy can evaluate the contribution of each input component to differentiate output categories.

In this paper, we propose an independent deep CNN (Szegedy et al., 2017) model to predict phenotypes from SNPs, which contains dual-stream of CNNs and can take either an imputed or non-imputed genotype matrix as the input. We also applied the saliency map deep learning visualization approach to select significant associated biomarkers from our trained model. To the best of our knowledge, this is the first study to apply a saliency map for a GWAS. The comparison results with traditional statistical methods (rrBLUP, Bayesian ridge regression (BRR), Bayesian A, and BL) and existing deep learning used several evaluation metrics on both simulation and experimental data, which indicate that our proposed deep learning model serves as a robust and efficient architecture in selecting germplasms and discovering genotype–phenotype relationships.

## Materials and Methods

### Dataset

We used an experimental soybean dataset and a simulation dataset as the benchmark to evaluate the performance of our deep learning model, as summarized in **Table 1**.

**Table 1:** Summary of soybean experimental dataset.

Dataset	Trait	Environment	Sample (*N*)	Heritability	Reference
SoyNAM	Yield	2013 Illinois	5,001	0.512	(Xavier et al., 2015)
	Protein	2012 Illinois	5,128	0.545	
	Oil	2012 Illinois	5,128	0.617	
	Moisture	2012 Illinois	5,128	0.582	
	Height	2013 Illinois	5,138	0.667	

**Soybean Dataset:** The soybean dataset from the soynam project was generated using a nested association panel (Xavier et al., 2015; Song et al., 2017). The soybean dataset contains more than 5,000 recombination inbred lines (Rils) and 4,236 common SNPs between imputed data and raw quality assured data. The genotype and phenotype data were available in the “SoyNAM” R Package (Xavier et al., 2015). We selected five traits from the 2013 and 2012 Illinois Location. Missing genotypes in the soybean dataset were imputed using the MaCH software (Li, et al., 2010) based on the HMM Approach. The imputation method applied on the soybean dataset was discussed in Xavier et al. (2016), who found it to have the best performance in imputing accuracy and phenotype predicting ability.

**Simulation Dataset:** The simulation dataset was constructed using Hypred (Technow, 2011), which simulates 10,000 F2 recombined individuals with 5,000 SNPs. We assigned quantitative trait locus (QTL) every 500 SNPs at SNP index position 100, 600, 1100, 1600, 2100, 2600, 3100, 3600, 4100, and 4600. No missing value was included in the simulation set.

The genotype matrix used as inputs for the three datasets was coded into 0, 1, or 2 to represent homozygous, heterozygous, and reference homozygous, respectively, and missing genotypes were coded as −1 for genotypes without imputation.

### Narrow-Sense Heritability

The narrow-sense heritability of each trait is calculated based on the BRR model from the R package SoyNAM. It is defined as the ratio of phenotypic variance due to additive genotypes as follows:

$$h^{2}=\frac{V_{g}}{V_{g}+V_{e}}$$

where V_g_ is the phenotypic variance and V_e_ is the residual variance estimated from a BRR model.

### Deep Learning Architecture

#### Genotype Coding With One-Hot

**Figure 1:**
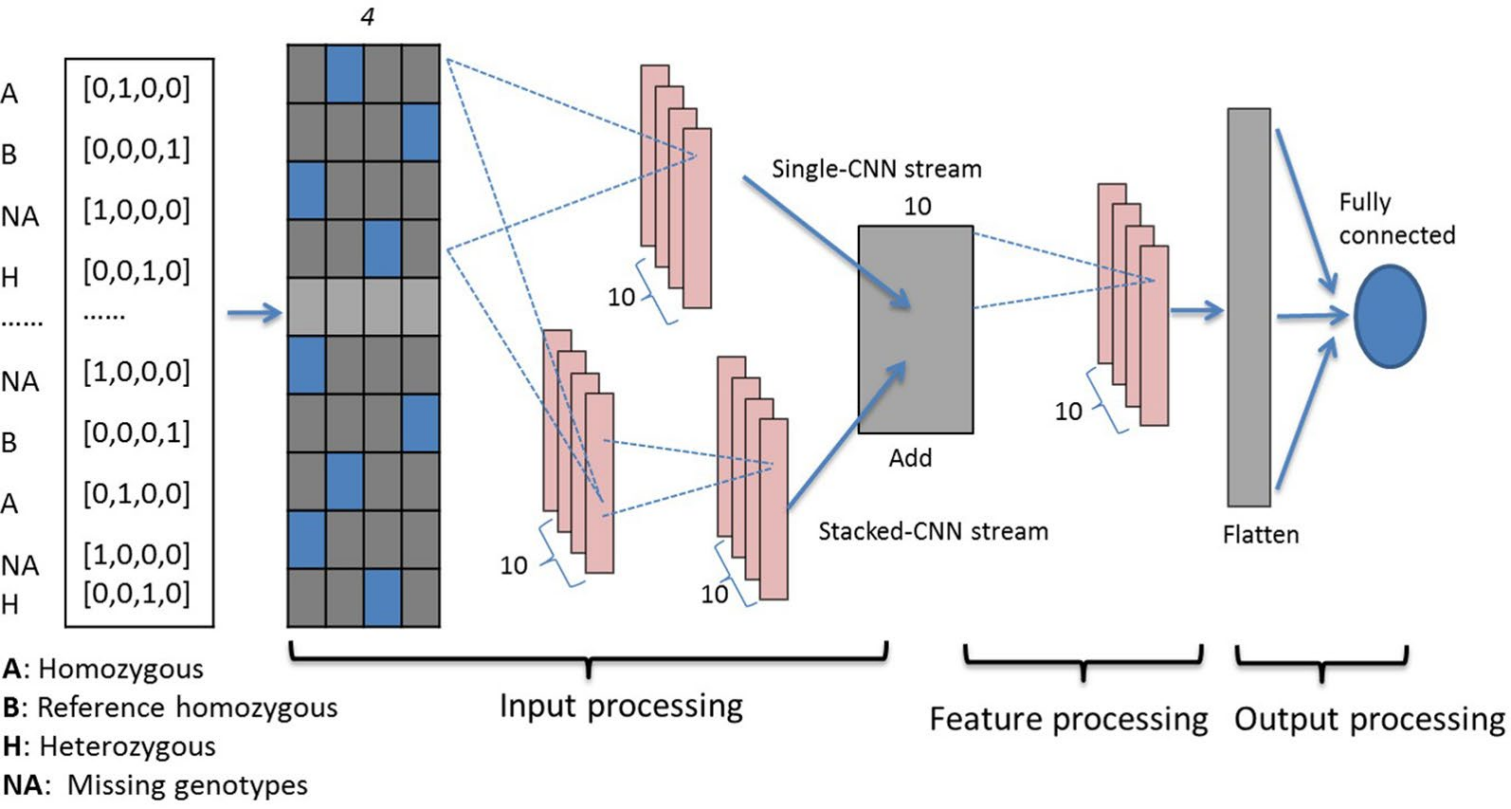
Dual-stream CNN model structure. Genotypes are one-hot coded and passed to the input processing block, which contains two streams of CNNs. The first stacked-CNN stream contains two feed-forward CNN layers with kernel sizes 4 and 20. The second single-CNN stream contains one CNN layer with kernel size 4, followed by an add-up layer to aggregate outputs from the two streams. The feature processing block contains another single convolution layer with kernel size 4. Processed features are then passed to the output processing block, which contains a flatten layer and a fully connected dense layer. CNN, convolutional neural network.

Three genotypes (0, 1, 2) and missing values (−1) are first encoded using one-hot binary coding and serve as the input vector. Using one-hot coding, each marker is represented by a four-dimensional vector with 1 at the index for one genotype and the rest of them are set at 0 as shown in the far left inset of **Figure 1**. For example, three genotypes [AA, Aa, aa] are represented as [0, 1, 0, 0], [0, 0, 0, 1], and [0, 0, 1, 0], respectively. The missing genotype is represented as [1, 0, 0, 0]. Encoded genotypes serve as input to our model.

#### Genotype Processing Blocks

Our dual-stream CNN-based deep network contains three building blocks as shown in **Figure 1**, i.e., the input processing block, the feature processing block, and the output processing block.

*Input Processing Block:* This block contains an input layer, a dual-CNN layer, which contains two parallel CNN streams (Szegedy et al., 2017) and a sum-up layer to combine the parallel CNN streams. The input layer contains one-hot encoded genotypes, and subsequently the encoded genomics makers are simultaneously passed to the dual-CNN layer. We applied the idea of residual learning (He et al., 2016) in this dual-CNN layer, which was first introduced for image recognition and classification to solve the vanishing gradient problem. The residual connection is a shortcut connection from a previous layer and was added to identity mapping used to form a residual mapping. This approach has been applied in predicting protein backbone torsion angles and protein contact maps (Wang S et al., 2017; Fang et al., 2018). In the dual-CNN layer, the single-CNN stream served as a residual connection to the other stacked-CNN stream. The stacked-CNN contains two stacks of 1D convolutional layer with different kernel sizes, 4 and 20; and the single-CNN stream contains one convolutional layer with kernel size 4. The sum-up layer is used to aggregate the outputs from previous dual-CNN layer, and it is the element sum of both.

In order to optimize the kernel sizes, we used the affinity propagation (AP) (Frey and Dueck, 2007) clustering method on the genotype features to help guide us in selecting convolution sizes in this block. AP divided genotypes into clusters without assigning a number of clusters. The algorithm estimates the cluster center as the “exemplar” from data points. Real-time messages were exchanged between data points until a set of exemplars and clusters emerges through minimizing negative Euclidean distance. This clustering algorithm has been applied in computer vision and regulates transcript gene identification (Vlasblom and Wodak, 2009). We conducted AP clustering on 4,236 SNPs from the soybean dataset and repeated the process 100 times. Python package “sklearn” was used for AP cluster estimates (Pedregosa et al., 2011). We recorded sizes of clusters from 100 runs and tested kernel sizes using the number of genotypes clustered together. We aimed to capture short-range and long-range marker effects at various scales across the genome (Xu and Taylor, 2009; Brodie et al., 2016) so that small and large convolving sizes were used in our model. We finalized 4 and 20 as our convolving kernel sizes for stacked-CNN stream and 4 for the single-CNN stream.

*Feature Processing Block:* After completing our work on the input processing block, we determined that the aggregated sim-up outputs with different kernel sizes had more powerful representations of important genotypes than with a single kernel size. Hence, another convolution layer with a small kernel size 4 was added to integrate all the outputs and to further process genotype features in this block.

*Output Processing Block:* After completing our work on the feature processing block, a flattened layer was added to convert the convolution layer into a flattened layer. The flattened layer integrates the extracted features from the previous feature processing blocks, and features are passed to the last dense output layer, which contains a single neuron to represent the final predicted phenotypes.

#### Activation Function

We used the inverse square root unit (ISRU) (Carlile et al., 2017) activation functions in the model, which is defined as follows:

$$Y=\frac{x}{\sqrt{1+ax^{2}}}$$

The ISRU function was applied to add constraint of the predicted phenotype value and to speed up the model learning process. The activation function is bound to the range $\left(-\frac{1}{\sqrt{a}},\frac{1}{\sqrt{a}}\right)$ . Thus, we estimated a according to the maximum observed absolute phenotype values, which are 0.5, 0.03, 0.02, 0.02, and 0.02 for grain yield, height, moisture, oil, and protein of the soybean dataset, respectively.

### Model Training for Overfitting Control

It is important for the deep learning model to avoid overfitting because of the small training population of our datasets and because the total sample size is much smaller than the number of genotypes used as features. To reduce the effect of overfitting, we added dropout layers (Srivastava et al., 2014) after convolutional layers with a dropout ratio of 0.75. We then applied the L2 regularization on the cost function of mean square error (MSE) between estimated and predicted phenotypes:

$$MSE={\frac{\sum_{i=1}^{i=n}\left(Y_{i}-Y_{i}^{'}\right)}{n}}^{2}$$

We also monitored the mean absolute error (MAE) on our validation set and stopped the model training process as soon as the observed MAE stopped decreasing enough to confirm cessation. Hyperparameters, such as batch size and learning rate, were tuned by Hyperas (Pumperla, 2019). The deep learning models were implemented using Keras 2.1.1 on a workstation with GPU NVidia GTX 1080 Ti.

### SNP Contribution Using Saliency Map

We defined saliency values based on the idea of saliency map (Simonyan et al., 2013) to measure individual marker effects and their associations with quantitative GWAS trait. In the phenotype prediction problem, saliency values can be interpreted as scores to indicate effects of markers inside a window at length of a decided convolution kernel size from our deep learning model. The saliency values can guide extracting meaningful SNPs that show high-order marker effects correlated with phenotypes. In our deep model, given a genotype matrix *X* (n*p) of *n* individuals and *p* genotypes, the phenotype value was estimated as follows:

Y≈WX+b

where *W* represents the trained weight of each genotype and *b* is the model bias. In this case, after training the model, we can retrieve the output from the last output layer and calculate gradients *w* with respect to each input genotype using independent testing set as below:

$$w=\frac{\partial (Y)}{\partial X}$$

Since our genotypes were coded into one-hot vectors with four dimensions as the model inputs, we define the saliency value of each genotype as the maximum absolute value of gradients among those four coding channels. Therefore, to calculate the saliency value SV of a single genotype whose index is *i* and is coded in the *c*-dimension of one-hot vector, we use the following function:

$$SV_{i}=MAX(ABS(w_{i,c}))$$

We then calculate the median saliency value of whole populations, and this population median value is used as a measurement of our SNP contribution.

### Model Performance With Cross-Validation

#### Phenotype Prediction Accuracy

To measure our dual-stream CNN deep learning model performance, we calculated the Pearson correlation coefficient (PCC) between genomic predicted phenotypes and observed phenotype values of the testing dataset. We compared our deep learning model with four statistical models (rrBLUP, BRR, Bayesian A, and BL) and three deep learning models using the same training, validating, and testing datasets. The rrBLUP was implemented using the “mixed.solve” function from the “rrBLUP” package (Endelman, 2011) based on the maximum-likelihood (ML) estimation. BRR, Bayes A, and BL were implemented using the “wgr” function from the “SoyNAM” package (Xavier et al., 2015) based on the Monte Carlo Markov chain (MCMC) strategy with 4,000 iterations and 500 burn-ins.

The three compared deep learning models were a dense network (Montesinos-López et al., 2018) using several dense layers, the deepGS (Ma et al., 2017) a feed-forward three layer convolutional neural work, and a single-stream CNN that only contains the stacked-CNN layers from our proposed model. Hyperparameters were adopted from published codes.

#### Snp Contribution Accuracy

To measure the performance of our saliency value associated with the genotype contribution, we compared our results with a standard GWAS method using “gwas2” function from “NAM” R package based on the empirical Bayesian model (Xavier et al., 2015) that the significance of each genotype marker was evaluated through the Wald statistical test value.

#### Ten-Fold Cross-Validation

All soybean individuals were first split into 10 equal folds, in which eight folds formed the training set. One fold was assigned as the validation set, and the remaining one fold was employed to test the model performance. We repeated the same process 10 times, and the average PCC from the 10 calculations was reported to measure model performance.

## Results and Discussion

### Model Performance and Comparison With Other Methods

#### Dual-Stream CNN Model Improves Performance on Low Heritability Phenotypes

By using deep learning, missing genotypes can be coded using the one-hot binary coding method and can be treated as a category of genotype through computation. We coded both raw and imputed genotype matrix with a one-hot vector with four channels and applied the same deep learning architecture on them. The comparison of average PCC using existing statistical and deep learning methods is shown in **Table 2**. Missing value is not accepted by statistical methods, and hence, we only show results of imputed genotypes of statistical methods. The singleCNN network has similar PCC to statistical methods, and our dual-stream CNN outperforms statistical model and singleCNN using same imputed genotypes. Among the five traits, PCC of trait yield increases from 0.41 to 0.43, moisture increase from 0.38 to 0.412 and oil increase from 0.388 to 0.412 that is better than height and protein increasing from 0.458 to 0.465 and from 0.392 to 0.402.

**Table 2:** Average Pearson correlation coefficient of five traits from cross-validation.

	Yield	Protein	Oil	Moisture	Height
dualCNN (imp/non-imp)	0.434/0.452	0.402/0.619	0.412/0.668	0.426/0.463	0.465/0.615
DeepGS (imp/non-imp)	0.347/0.391	0.231/0.506	0.344/0.531	0.024/0.310	0.357/0.452
Dense (imp/non-imp)	0.359/0.449	0.357/0.603	0.401/0.657	0.370/0.427	0.434/0.612
singleCNN (imp/non-imp)	0.422/0.463	0.380/0.573	0.392/0.627	0.370/0.449	0.442/0.565
rrBLUP	0.412	0.392	0.39	0.413	0.458
BRR	0.422	0.392	0.39	0.413	0.458
Bayes A	0.419	0.393	0.388	0.415	0.458
Bayesian LASSO	0.419	0.394	0.388	0.416	0.458

CNN, convolutional neural network; BRR, Bayesian ridge regression.

Compare to singleCNN, performance of proposed dualCNN increases by adding a parallel single-CNN stream to the stacked-CNN stream. The add-up layer then integrates feature maps from both CNN streams, and this is necessary due to the loss of important features through convolving process with different kernel sizes, and it strengthens the signal of genotype features.

#### Predicting Phenotype With Imputed vs Non-Imputed Genotype Using Deep Learning

All four deep learning based methods have higher PCC on non-imputed than imputed genotypes (**Table 2**). The soybean dataset has ∼25% missing genotypes in the quality assured raw datasets. One reason deep learning model has higher predicting ability on raw datasets may be because the imputation process fills most missing genotypes with reference alleles, and it deflates the effects of different genotypes. Imputation methods assimilate missing genotype effects based on non-missing genotypes, which may compromise the prediction ability of selected quantitative traits.

**Figure 2:**
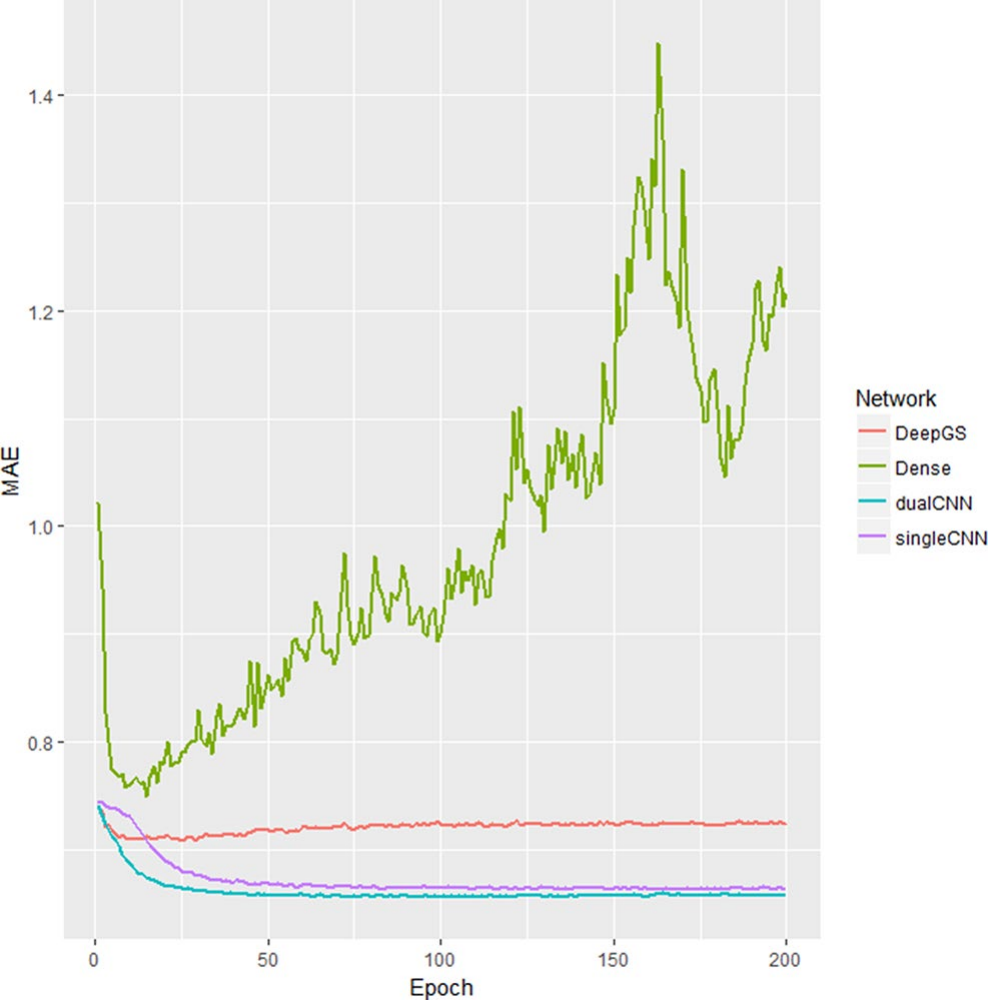
Training loss different deep learning models. The x-axis is number of epochs; the y-axis is the training the loss of mean absolute error (MAE) of validation dataset. The singleCNN (purple), dualCNN (blue), and Dense (green) network are conserved, and DeepGS is overfitting after 20 epochs, and our dualCNN has the lowest training loss. CNN, convolutional neural network.

Our dualCNN outperforms single-stream CNN and followed by a dense network (Montesinos-López et al., 2018) and then the DeepGS (Ma et al., 2017) for this soybean dataset (**Figure 2**) with lowest training loss on validation set. DualCNN, singleCNN, and the dense network have close performance on high heritability traits of oil and height, and our dualCNN has better performance in the other three low heritability traits yield, protein, and moisture on both imputed and non-imputed dataset. The dense network is better than deepGS for this soybean dataset, probably because the deepGS with more parameters is easier to be over-trained than the dense network. The DeepGS has a convolution layer of kernel size 18 that is not fit for the soybean SNP distribution of whole genome, while the dense network does not contain convolution layer, and each SNP was treated as a feature contribute independently to associated phenotype. But this dense network may also fail to integrate neighbor SNP associations within the convolution kernel.

### Effects of Training Population on Model Performance

**Figure 3:**
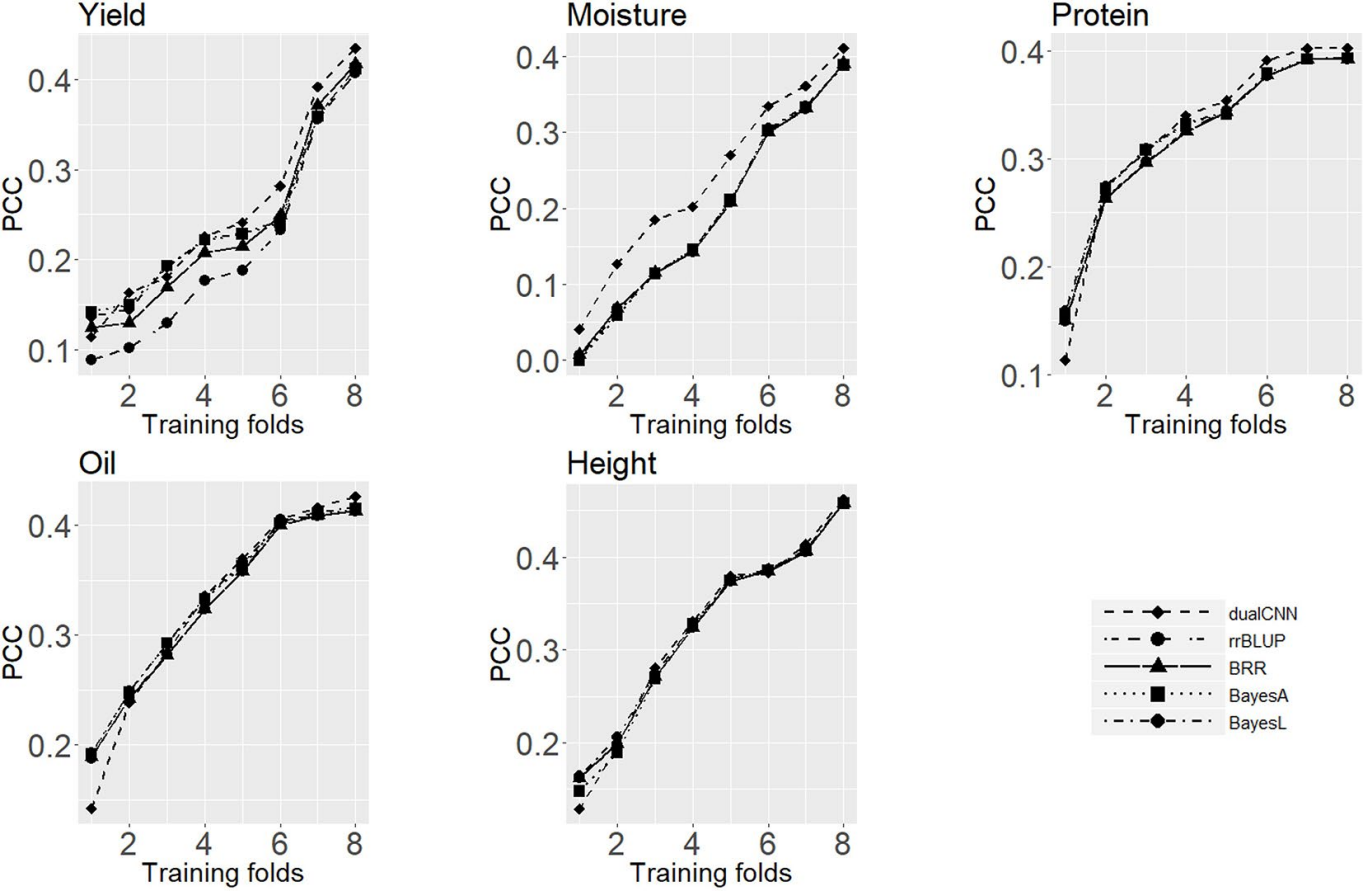
Average Pearson correlation coefficient of five traits using different sizes of training dataset. The x-axis is number of folds of training data; the y-axis is the average Pearson correlation coefficient from cross-validation.

The training population size is a major factor in both machine learning and statistical approaches, and it directly affects predicting performance (Xavier et al., 2016;Cericola et al., 2017). Good training data will be able to represent the whole population structure and to satisfy the prior assumption of genotype effects for statistical models. **Figure 3** shows the average PCC of five traits predicted on the testing set trained with different sizes of training sets. For soybean dataset, the dualCNN reaches a higher PCC than the other four statistical models and was less affected by the training population size in low heritability traits as yield, moisture, and protein. As long as the training size reached 1,500, our model showed a higher performance than statistical models. The whole genome regression (BRR, BayesA, and BayesLASSO from the NAM package) had a better performance than the rrBLUP package, since the former applies Gibbs resampling and MCMC to update regression coefficients.

### Comparison of Genotype Contribution Between Saliency Map and GWAS

**Figure 4:**
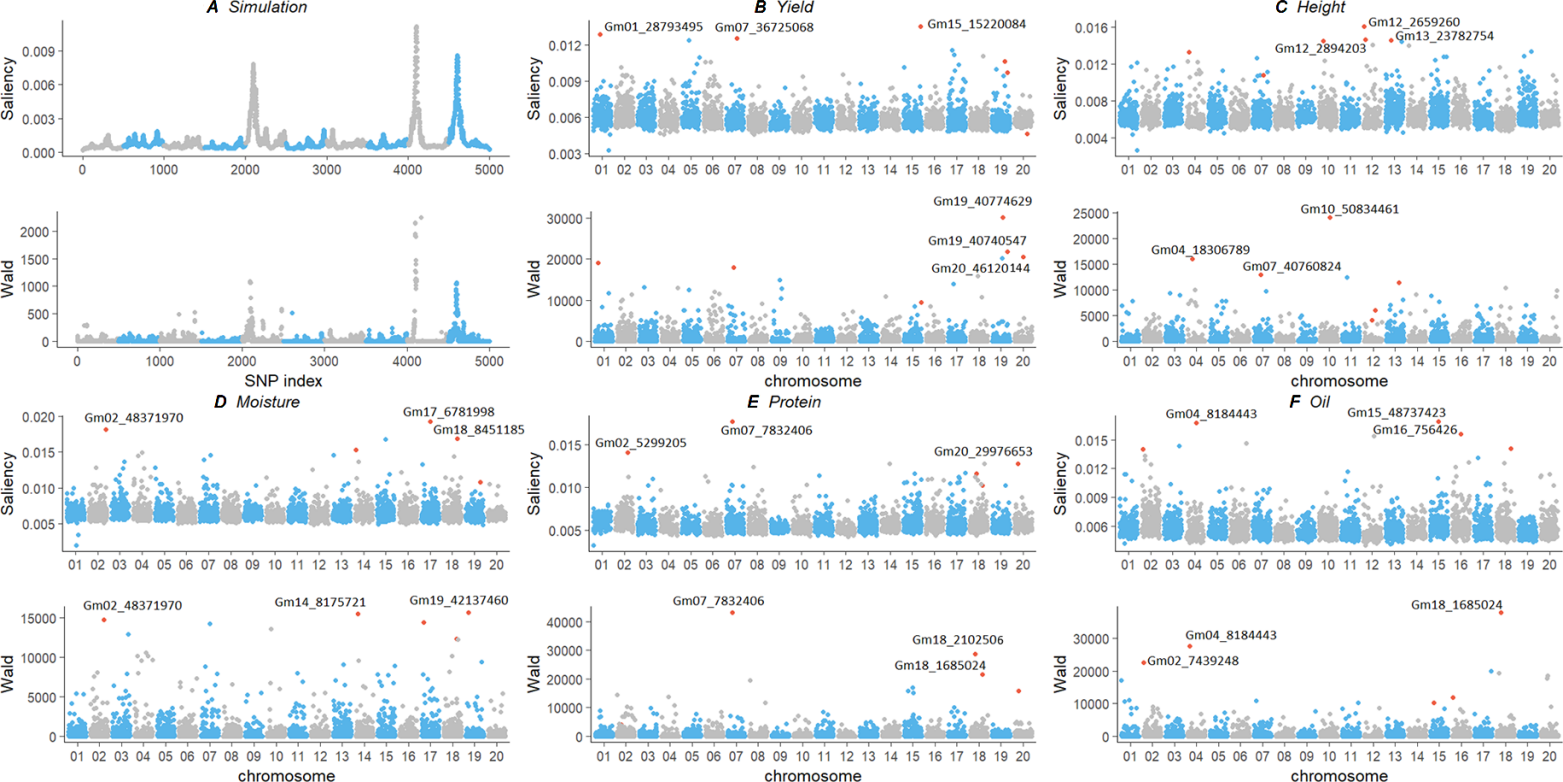
Comparison of genotype contribution using saliency map and GWAS Wald test of simulation **(A)** and experimental soybean dataset with five traits **(B**–**F)**. The x-axis is the index of SNPs in the genotype matrix; the y-axis is the saliency and Wald test results. Top ranked SNPs were plotted in red. GWAS, genome-wide association study; SNP, single-nucleotide polymorphism.

We compared our deep learning saliency value against GWAS results through Manhattan plot using a simulation and an experimental dataset (**Figure 4**). Their calculated saliency values and Wald test score are available at **Supplemental Table 1**. For the two datasets, we observed a similar curve pattern from both saliency values and the GWAS Wald test score. In the experimental dataset, we compared the top three SNPs according to their significance and discussed potential markers discovered using our method. The top ranked SNPs and their relative position in the other measurement were plotted in red. Since the soybean linkage disequilibrium extent region of a significant SNP ranges from ∼20 to ∼100 kb, we located the closest gene within the 20-kbp region centered with the identified SNPs and annotated genes with Gene Ontology (GO) (Ashburner et al., 2000), protein family (PFAM) (Bateman et al., 2004) using Soybase Gbrowser (Grant et al., 2009) and SoyKB (Joshi et al., 2012;Joshi et al., 2013) according to gene model “Glyma.Wm82.a1.v1.1” (Schmutz et al., 2010). Gene annotations and literature reports indicate those markers, and their nearby regions are highly associated with their traits. Several novel markers and regions were detected and are listed as follows:

*Simulation:* Both saliency values and GWAS results showed the same three peaks on the simulation dataset in **Figure 4**. The three peaks were correlated with the QTLs assigned at the SNP index positions of 2100, 4100, and 4600. It strongly indicates that the saliency approach can find similar SNPs with statistical GWAS models.

*Grain Yield:* For soybean grain yield, we identified SNPs Gm01_28793495, Gm07_36725068, and Gm15_15220084, with the highest saliency value as shown in **Figure 4**. The top SNPs from GWAS, Gm19_10774629, and Gm19_40740547 also have high saliency value and locate in the same haplotype block with a linkage disequilibrium *r*^2^=0.9766. Potential genes Glyma15g18430 and Glyma15g18450 are close to SNP Gm15_15220084. Glyma15g18430 reported by Won Oh et al. (2014) has differentially changed soybean root proteins with gibberellic acid treatment under flooding stress. It belongs to the glycosyl hydrolases family (PF01301) and involves in carbohydrate metabolic process (GO: 0005975). Glyma15g18450 is associated with plant flowering (Jung et al., 2012) has biological process of flower development (GO: 0009908) and leaf morphogenesis (GO: 0009965).

*Plant Height:* For soybean plant height, saliency and Wald test value were plotted in **Figure 4**. One region on chromosome 12 is most significant from the saliency value but not present in the GWAS results; thus, we investigated the closest gene, Glyma12g04400, of the SNP Gm12_2894203. This gene belongs to the putative snoRNA binding domain (PF01798,GO:0003677) and is reported by Komatsu et al. (2012; 2014) with differential protein change under flooding stress. The region around SNP Gm12_2659260, from 26624**kb to 26629**kb, is reported in a 302 resequencing soybean dataset (Zhou et al., 2015) as a copy number variation signal that is associated with plant height. Two SNP Gm12_2894203 and Gm12_2659206 are in the same haplotype block with *r*^2^=0.9510. The closest region of SNP Gm13_23782754 is reported as a QTL region associated with plant height (Zhang X et al., 2018). Both saliency and GWAS identified SNP Gm04_18306789 and Gm10_50834461, and close gene Glyma10g44500 is associated with salt tolerance (Pantalone et al., 1997) and is involved in lipid transport (GO: 0006869).

*Moisture:* The most significant SNP Gm17_6781998 and Gm18_8451185 from saliency values also present in the GWAS results in **Figure 4**. The closest gene Glyma17g09165 belongs to the protein kinase domain (PF00069) and is involved in the biological process in response to cold, wounding, salt stress, and mannitol stimulus, that is, GO: 0009409, GO: 0009611, GO: 0009651, and GO: 0010555, respectively. Gene Glyma18g09550 belongs to seed storage family (PF00234) with lipid transport (GO: 0006869). Both methods identified SNP Gm02_48371970, and the closest gene Glyma02g43602 is response to fungus, chitin, and fatty acid (GO: 0009620, GO: 0010200, GO: 0071398).

*Protein:* For the soybean protein content, saliency value and Wald test score were plotted in **Figure 4**. The SNPs Gm02_5299205 and Gm20_29976653 are only present in the saliency value, and the former is in gene region of Glyma02g06650. The region around both SNPs may associated with protein content in chromosome 2 (Akond et al., 2012) and chromosome 20 (Hwang et al., 2014). Both saliency value and Wald test score indicate SNP Gm07_7832406 as the most significant one, and it is a missense mutation in the coding sequence region of gene Glyma07g09400. This gene belongs to the PP-loop family (PF01170) with molecular functions of ATP binding, ligase activity, and forming carbon–nitrogen bonds (GO: 0000166, GO: 0005524). This could also be a new marker associated with protein QTL region (Jun et al., 2008).

*Oil:* For SoyNAM protein content, saliency value identified a potential novel SNP Gm15_48737423, and it is inside the gene region on Glyma15g41600 **Figure 4**. It belongs to the pyridocal-phosphate-dependent enzyme protein family (PF00291) and involves a sulfur amino acid metabolic process, a cysteine biosynthetic process, and a cell wall modification (GO:0000096, GO: 0006535, GO: 0042545). This gene was reported by Prince et al. (2015) with an association with potential root QTL, and it was also reported as a putative β-substituted alanine synthase isoform by Yi et al. (2010). A new marker around region Gm16_756426 also detected associated with oil content (Jun et al., 2008). The common SNP Gm04_8184443 is close to gene Glyma04g09900, and this gene belongs to the protein tyrosine kinase family (PF07714), which involves the protein phosphorylation process and the oligopeptide transport process (GO: 0006468, GO: 0006857).

## Summary

In this paper, we proposed a deep learning of dual-stream CNN method to accurately predict phenotypes using SNP markers that can avoid missing genotype imputation. We also proposed using saliency map approach to measure SNPs associated with the selected traits, which helps to determine important markers and QTL regions. We have explored several different deep learning architectures, such as the fully connected DNN, deepGS, single-stream CNN, as well as several statistical approaches. We have found the two-stream CNN structure has best predicting performance on real experimental datasets, especially with low heritability quantitative traits, and it less relies on the structure of training population. To our knowledge, we are the first to use saliency value as a measurement of SNP contribution. By using CNN, the saliency map calculates the genotype effect not only as a single marker but also through convolving with their neighboring SNPs, which helps detect important trait associated regions.

Computing efficiency is also important for machine learning problems. It may not be fair to compare computing efficiency of a deep learning model applicable on GPU with statistical models on CPU, but GPU-based deep learning models actually outperformed most R-based genomics selection packages with much less computing time. Our dual-stream CNN model costs around 10 minutes, and statistical regressions cost more than 3 hours to train the model and test results for the soybean dataset. Taking the advantage of GPU computing and progress in the state-of-art deep learning technique, we expect this deep learning approach to be useful in accurately predicting phenotypes and detecting meaningful genomic markers in a more efficient way. In the future, we will continue improving our model and studying effects of genotype interactions on phenotypes explicitly. We will also work with biologists to interpret underlying biological significance of the prediction results. It is recommended to use deep learning on a large population of high-dimensional genotype and low-heritability phenotypes in phenotype prediction and biomarker selection.

## Data Availability Statement

The deep learning model, results, and datasets used can be found at https://github.com/kateyliu/DL_gwas.

SoyNAM dataset can be found at https://cran.r-project.org/web/packages/SoyNAM/index.html.

## Author Contributions

YL: designing the experiments, modeling, summing up, and writing the manuscripts. FH and DW: performing discussing and revising experiments. JW: generating simulation data. TJ and DX: advising and revising the project.

## Funding

This work was partially supported by National Institutes of Health (award R35-GM126985).

## Conflict of Interest

The authors declare that the research was conducted in the absence of any commercial or financial relationships that could be construed as a potential conflict of interest.

## Supplementary Material

Supplemental Table1 Each column in the table represents: SNP: SNP ID wald_protein: Wald test value of protein sigma2_protein: estimated residual variance of protein eff_protein: estimated allele effect of protein saliency_protein: saliency value of protein wald_yield: Wald test value of yield sigma2_yield: estimated residual variance of yield eff_yield: estimated allele effect of yield saliency_yield: saliency value of yield wald_oil: Wald test value of oil sigma2_oil: estimated residual variance of oil eff_oil: estimated allele effect of oil saliency_oil: saliency value of oil wald_height: Wald test value of height sigma2_height: estimated residual variance of height eff_height: estimated allele effect of height saliency_height: saliency value of height wald_moisture: Wald test value of moisture sigma2_moisture: estimated residual variance of moisture eff_moisture: estimated allele effect of moisture saliency_moisture: saliency value of moisture.
